# Controlling Experimental Parameters to Improve Characterization of Biomaterial Fouling

**DOI:** 10.3389/fchem.2020.604236

**Published:** 2020-12-11

**Authors:** Alexander H. Jesmer, Ryan G. Wylie

**Affiliations:** ^1^Department of Chemistry and Chemical Biology, Hamilton, ON, Canada; ^2^School of Biomedical Engineering, McMaster University, Hamilton, ON, Canada

**Keywords:** biomaterials, low-fouling, protein interactions, cell adhesion, non-specific binding

## Abstract

Uncontrolled protein adsorption and cell binding to biomaterial surfaces may lead to degradation, implant failure, infection, and deleterious inflammatory and immune responses. The accurate characterization of biofouling is therefore crucial for the optimization of biomaterials and devices that interface with complex biological environments composed of macromolecules, fluids, and cells. Currently, a diverse array of experimental conditions and characterization techniques are utilized, making it difficult to compare reported fouling values between similar or different biomaterials. This review aims to help scientists and engineers appreciate current limitations and conduct fouling experiments to facilitate the comparison of reported values and expedite the development of low-fouling materials. Recent advancements in the understanding of protein–interface interactions and fouling variability due to experiment conditions will be highlighted to discuss protein adsorption and cell adhesion and activation on biomaterial surfaces.

## Introduction

*In vitro* biofouling characterization is crucial for the discovery of materials for medical implants and other blood or tissue contacting devices. Inaccurate or incomplete biofouling characterization may hinder the discovery of promising biomaterials as initial results may not translate to *in vivo* tests (Ratner, 2019). Because of the difficult nature of executing comparable biofouling experiments between the ever-expanding library of biomaterials and many different procedures and techniques, data comparison between reported values remains difficult or impossible (Heggestad et al., 2019). The following will therefore discuss the unwanted biological outcomes from biofouling, interactions of proteins and cells with material interfaces as a function of experimental conditions, and fouling characterization techniques, all with respect to prominent biomaterial applications.

Surfaces of implanted and biofluid contacting materials are subject to interactions with biological macromolecules, cells, and tissues (Anderson et al., 2008). Left uncontrolled, these interactions can lead to deleterious inflammatory responses, infections (Busscher et al., 2012), implant failures (Trindade et al., 2014), and loss of material performance. Protein interactions with biomaterial surfaces can lead to thrombus formation (Gorbet and Sefton, 2004), degradation of performance (Xie et al., 2018), and cell adhesion, where the identity (Swartzlander et al., 2015; Vu et al., 2019) and state of adsorbed proteins dictate downstream cell responses (Veiseh and Vegas, 2019).

Host mammalian cells can encapsulate, or fibrose, the implanted material leading to poor integration, loss of function in implanted sensors, or drug delivery vehicles or degrade the biomaterial itself via reactive oxygen species (Welch et al., 2020). For example, fibrotic responses to implanted pacemakers can lead to fatal outcomes in nearly 2% of removal procedures despite advances (Rennert et al., 2014) and can degrade hearing and cause device failure in 1% of cochlear implants (Foggia et al., 2019).

Bacterial adhesion to biomaterials can lead to persistent and difficult-to-treat infections through the production of biofilms, which increases antibiotic resistance, as well as the failure of implants altogether (Arciola et al., 2018). With respect to bacterial complications, approximately two catheter-associated urinary tract infections occur every 1,000 days of catheter use, contributing to urinary tract infections being the most common hospital acquired infection (Saint et al., 2016). Furthermore, there are 100,000–200,000 central line–associated blood infections per year in the United States, even after the widespread implementation of guidelines to reduce infections over the last two decades (Bell and O'Grady, 2018).

**Figure 1:**
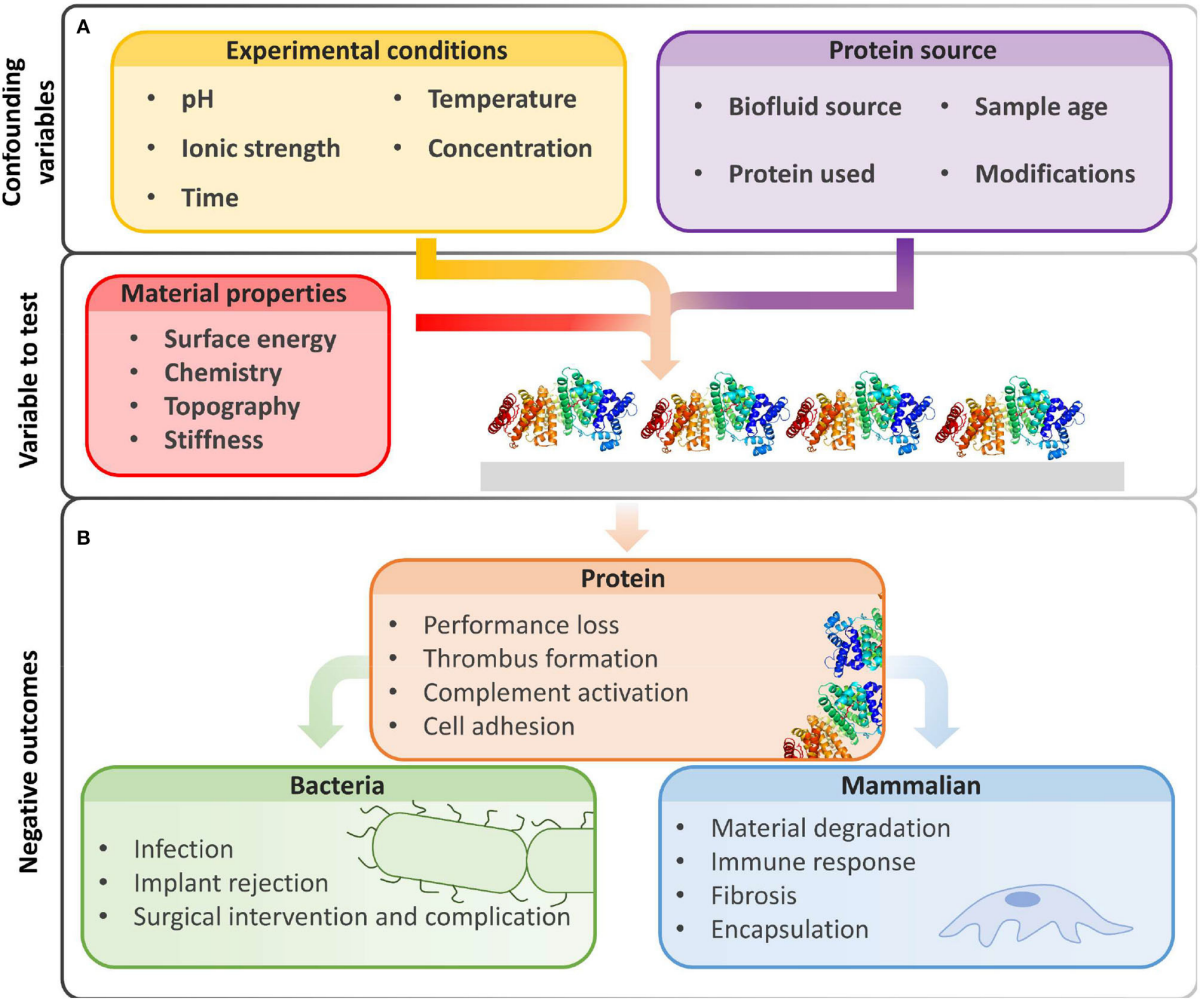
Beyond intrinsic material properties, experimental conditions and biological reagents greatly influence fouling results. **(A)** The amount of non-specific protein adsorption on biomaterial surfaces is influenced by experimental conditions and protein sources chosen by the user. **(B)** Biofouling may hinder device performance and produce unwanted biological events due to non-specific protein adsorption, increased bacterial adhesion, and deleterious interactions with mammalian cells. Image of PDB ID 1BJ5 (Curry et al., 1998) created with (PyMol, 2015).

To control (Vishwakarma et al., 2016) non-specific biomolecule adsorption or cellular interactions with material interfaces and identify promising low-fouling materials, we must first select appropriate conditions for *in vitro* fouling experiments. For non-specific adsorption and adhesion measurements, we must carefully select the protein or biofluid source, experimental conditions, and characterization methods, as they all influence protein adsorption and cell adhesion (Figure 1). Because of the great number of variables, it is often difficult to compare reported results between experiments (Heggestad et al., 2019). It is therefore useful to include controls and highlight limitations to prevent problematic comparisons.

Ultimately, the material and intended application should guide the selection of biofouling experiments. As best as possible, *in vitro* testing should recapitulate the biological conditions, and the influence of any deviations should be considered. The characterization tool will depend on intrinsic material properties and the biological environment needed for the experiment. The following will provide a brief overview of factors that influence protein and cell adhesion on biomaterial surfaces and cover common biofouling characterization methods.

## Non-Specific Adsorption and Adhesion

### Protein Adsorption

The interaction of proteins with surfaces is common in biological environments, and their understanding is required to design low-fouling biomaterials. Controlled and specific protein–surface interactions are essential for non-medical and medical devices for implant integration and tissue engineering (Fernandez-yague et al., 2015). Protein–surface interactions can also be harmful; they may prevent integration with host tissue for functional recovery, as well as promote harmful blood clots and immune responses (Vishwakarma et al., 2016).

Proteins adsorbing to biomaterial surfaces displace water molecules (Chen et al., 2010) and interact with the material through non-covalent Van der Waals, hydrogen bonds, electrostatics, and hydrophobic interactions (Norde, 1996). Proteins can then unfold or rearrange on the surface at different rates (Roach et al., 2005). This process of adsorption, conformational change, and displacement may be dynamic and competitive. For example, the Vroman effect describes the competitive displacement of abundant and high-mobility proteins with proteins that have higher surface affinity and lower mobility (Vroman et al., 1980). The rate of displacement is dependent on protein concentration and material properties (Horbett, 2018).

Because of limitations in experimental techniques, quantification of protein adsorption cannot always be conducted under physiological conditions. However, any deviations from physiological conditions may change the amount of protein adsorption (Figure 1A). Experimental factors that alter protein interactions with materials include (1) protein concentration and protein source (e.g., solution vs. serum), (2) charge screening by ionic strength or pH changes, (3) fluorescent labels that increase hydrophobicity, and (4) temperature.

The concentration of protein in solution impacts the amount of non-specific protein adsorption on surfaces (Chandrasekaran et al., 2013; Hedayati et al., 2020). Protein concentration can also influence protein stability and rates of unfolding and surface displacement, leading to dynamic changes in adsorbed protein populations (Hedayati et al., 2020). For example, increasing the concentration of milk or blood-sourced proteins from 0.1 to 2 mg mL^−1^ led to greater adsorption on stainless steel (Chandrasekaran et al., 2013). At lower concentrations, the trend may be reversed; fibrinogen and albumin adsorption was greater with ~1 μg mL^−1^ than 1 mg mL^−1^ solutions (Hedayati et al., 2020). The wide variation of utilized protein concentrations also complicates comparisons. As determined through observations and a selection of reports, protocols without fluorescent or radioactive proteins typically use concentrations of 1 (Dong et al., 2016; Yu et al., 2017) to 80 mg mL^−1^ (undiluted blood serum), whereas protocols with fluorescently labeled proteins regularly use 0.1–10 mg mL^−1^ (Sundaram et al., 2011; Liu et al., 2017; Yang et al., 2018; Feng et al., 2019), ranges that may be well below or above physiological concentrations.

The amount and identity of adsorbed proteins can also change with biofluid source; even pooled blood samples vary in protein adsorption (Pereira et al., 2014). Age of biofluid samples modifies protein conformation, leading generally to higher levels of non-specific adsorption (Yang et al., 2009). Fluorescent labels on protein surfaces may influence the orientation of proteins on surface, modifying downstream cell adhesion. Finally, adsorbed proteins may be displaced by other proteins over time when using complex protein solutions, which introduces time as a variable.

### Cell Adhesion to Protein Covered Surfaces

**Figure 2:**
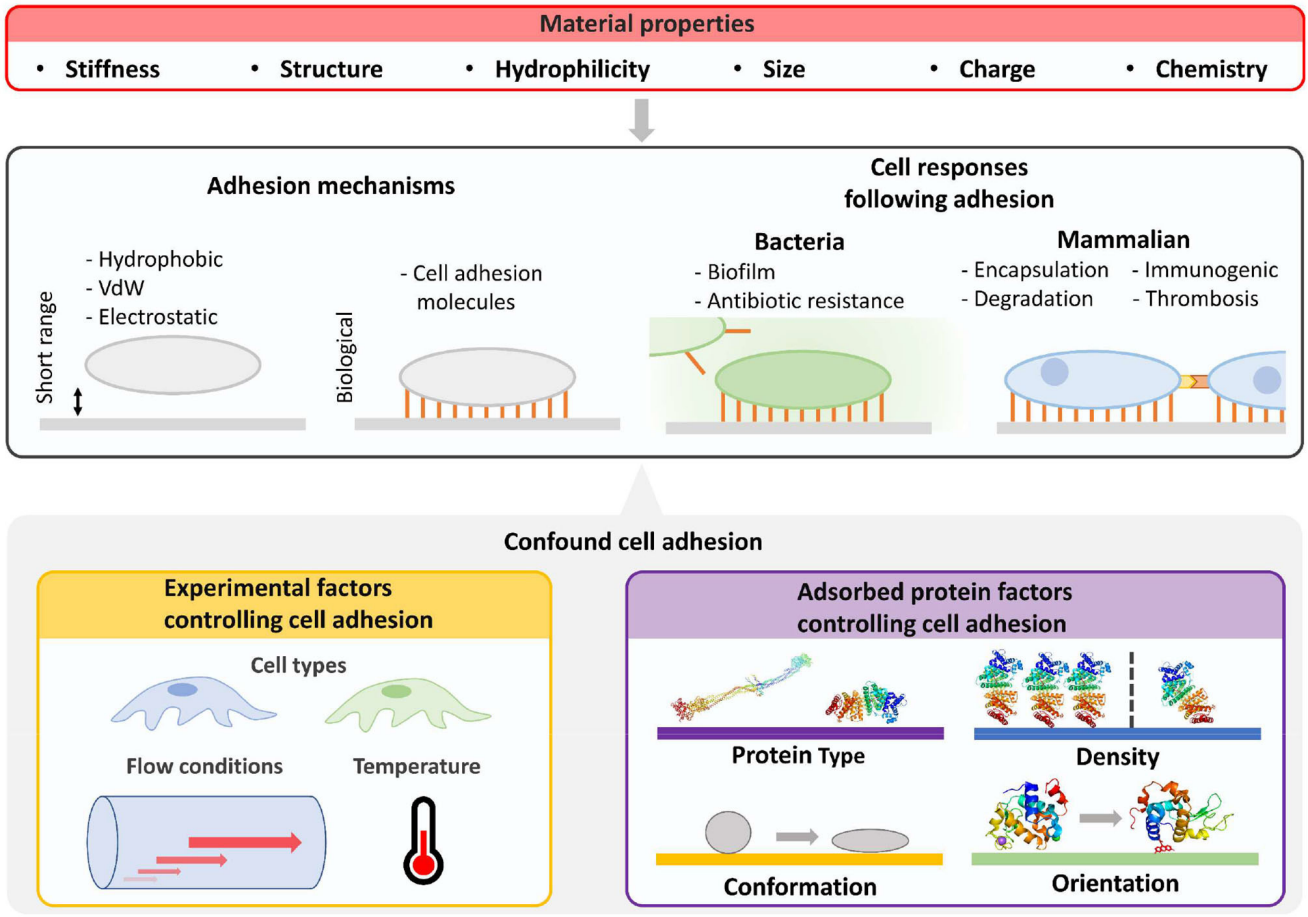
Characterization of cellular fouling due to differences in material properties is confounded by experiment factors and properties of adsorbed surface proteins. Beyond material properties, the number and strength of surface adhered cells will be determined by cell type, flow conditions, and properties of adsorbed proteins such as type, density, orientation, and conformation. Cell responses will then be influenced by altering biofilm formation for bacteria and potential immune responses in mammalian environments. Furthermore, biologically mediated material degradation may occur. Images of PDB IDs 1EI3 (Yang et al., 2000), 1BJ5 (Curry et al., 1998), 5WRA (Sugahara et al., 2017), and 5IEL (Kachalova et al., 2017) created with (PyMol, 2015). VdW, van der Waals force.

Cell adhesion to surfaces with adsorbed proteins is controlled by protein identity, density (i.e., ng cm^−2^), conformation, and orientation (Felgueiras et al., 2018) (Figure 2). For non-adhesive proteins, proteins partially or fully denatured may result in greater cell or bacteria adhesion by modifying surface hydrophobicity. For adhesive proteins, the folded protein will likely result in greater mammalian cell adhesion due to cell surface integrins (Anselme et al., 2010). For example, only ~10 ng cm^−2^ of fibrinogen is required for most cells to adhere, and even less for monocytes (Shen et al., 2001). Beyond the protein coating, adhesion is also dependent on surface biomaterial properties, cell cycle stage, and environmental factors such as pH.

Cell adhesion to surfaces is also driven by material properties independent of protein adsorption (Rahmati et al., 2020) (Figure 2). Surface stiffness can control the adhesion of cells *in vitro* (Yeung et al., 2005) and influence cell signaling and differentiation *in vivo* (Miller and Davidson, 2013). Surface structure, roughness, and engineered structures can also influence cell adhesion (Graham and Cady, 2014), whereas patterns direct cell alignment (Nguyen et al., 2016). Selective cell adhesion has been demonstrated by controlling feature size on surfaces. For example, surface features smaller than the footprint of mammalian cells can prevent non-specific adhesion of osteoblasts and promote bacterial adhesion (Wu et al., 2011).

### Potential Unwanted Events Due to Cell Adhesion

Because protein adsorption onto low-fouling materials is often below detection limits, quantifying cell adhesion to surfaces may be necessary to contextualize protein fouling data and investigate potential cell-based biofouling events. Host cell interactions with biomaterials may result in a range of biochemical processes such as macrophage activation [and the foreign body response (FBR)], platelet activation, and thrombus formation. The FBR is initiated by protein adsorption followed by monocyte recruitment and differentiation into macrophages, formation of giant cells, and fibroblast recruitment for the formation of fibrotic capsules (Veiseh and Vegas, 2019). In blood-contacting materials, platelet and leukocyte adhesions are part of a complex cascade that leads to thrombosis with potentially fatal effects (Gorbet and Sefton, 2004). The *in vitro* evaluation of blood contacting materials has been well reviewed recently and is beyond the scope of this manuscript (Weber et al., 2018).

Following bacterial adhesion to a surface, some bacteria can form biofilms that may lead to serious infections. Biofilms are extracellular matrices of proteins and carbohydrates that contain bacteria colonies with distinct environments (Flemming et al., 2016). Bacteria in biofilms are less susceptible to antibiotics, making infections difficult to eliminate; antibiotics may be up to 1,000-fold less effective against sessile than planktonic bacterial states (Stewart, 2015). To initiate biofilm formation, sufficient bacterial load must be present at the surface; load requirements depend on biological conditions and species. Biofilm characteristics are predominantly determined by the environment; temperature and nutrient availability are primary drivers of growth; shear forces control biofilm thickness and density. In some cases, biofilms are induced by shear forces, and still conditions hinder biofilm formation (Weaver et al., 2012).

## Typical Proteins and Cells Used for Fouling Experiments

Several types of proteins and cells are routinely used to characterize biomaterial fouling. For proteins, individual or mixtures are used to either reflect the most abundant proteins in human serum and extracellular environments (e.g., human serum albumin) or highlight important downstream processes such as cell adhesion (e.g., fibronectin) or thrombus formation (e.g., fibrinogen) (Table 1). Mixtures of proteins have been used to mimic abundant circulating proteins (Fabrizius-Homan and Cooper, 1991) or to study the Vroman effect (Goor et al., 2017). Cell types for characterization should be selected to elucidate biomaterial responses relevant for the intended application or implant-related infections (Table 1).

**Table 1:** Biological materials commonly used for biofouling characterization with respect to endpoint measurement.

	**Biological material**	**Primary reason for choice**	**End-point measurement**	**References**
Proteins	Albumin	Abundance in serum and blood	Non-specific adsorption	Brash et al., 2019
	Fibrinogen	Role in thrombosis	Non-specific adsorption, conformation bioactivity	Horbett, 2018
	Fibronectin	Role in cell adhesion		Parisi et al., 2020
	Vitronectin	Role in cell adhesion		Wilson et al., 2005; Franz et al., 2011
Biofluids	CSF	Biomaterials for central nervous system	Amount and profile of adsorbed proteins	Harris et al., 2011
	Serum	Typical protein source in cell culture and blood		Gunkel and Huck, 2013
	Whole blood	Required for testing compatibility of blood contacting materials	Adsorption, complement activation, thrombosis	Weber et al., 2018
Bacteria	*E. coli*	Biotechnology model organism, implicated in some implant associated infections	Adhesion	Francolini et al., 2017
	*P. aeruginosa*	Common bacteria implicated in hospital acquired infections, forms biofilms	Adhesion, biofilm formation	Wagner et al., 2016
	*S. epidermidis/S. aureus*	Responsible for over 50% of implant infections		Oliveira et al., 2018
Mammalian	Fibroblast	Lead to fibrosis following chronic inflammation	Adhesion	Witherel et al., 2019
	Macrophage	Implicated in immune responses to material	Adhesion, polarization	Huyer et al., 2020

*CSF, cerebrospinal fluid; E. coli, Escherichia coli; P. aeruginosa, Pseudomonas aeruginosa; S. aureus/S. epidermidis, Staphylococcus aureus/Staphylococcus epidermidis*.

## Methods for Quantifying Bulk Protein Fouling on Planar Surfaces

A number of quantitative methods are commonly employed to measure protein adsorption on low-fouling coatings using sensors, planar surfaces, or detection labels and reagents. Because of differences in detection, fouling results from different techniques and methods must be carefully examined before comparison. Each technique will introduce deviations in experimental parameters that influence fouling levels such as concentration, flow, temperature, and time. Furthermore, many techniques use sensors or surfaces that are coated with low-fouling materials; grafting efficiency will therefore further introduce variability. Additionally, it has recently been shown that minor deviations during sample preparation for characterization techniques can also impact fouling levels (Visova et al., 2020). To overcome these differences in sample preparation, as well as reduce variability between samples, several techniques for characterizing low-fouling coatings have been extended to high-throughput screening (HTS) (Hook et al., 2010, 2012; Magennis et al., 2016).

Several of these methods provide quantitative data without the need for fluorescent or radioactive labels, allowing for biofouling characterization with a wide variety of proteins and complex biofluids (Table 2), whereas techniques that require fluorescent or radioactive labels on proteins are generally limited to biofouling experiments with individual proteins.

**Table 2:** Advantages and limitations of commonly employed techniques to characterize protein adsorption and cell adhesion.

	**Technique**	**Advantages**	**Limitations**	**Label required**	**LOD**	**References**
Protein adsorption	SPR	Good detection limit	Substrate must be planar, thin coatings, limited sensors options	–	0.3 ng cm^−2^	Blaszykowski et al., 2012
	QCM-D	Good detection limit, sensitivity to viscoelasticity	Planar, thin materials, stringent substrate materials	–	1.8 nm cm^−2^	Edvardsson, 2009
	Ellipsometry	Good detection limit	Specific material requirements	–	0.1 nm^a^/5 ng cm^−2^	Richter and Brisson, 2005
	TIRF	Single molecule, time dynamic	Planar, label required, low concentration limit	+	—	Hedayati et al., 2020
	XPS	Elemental and bonding information	Qualitative, presence of high background signals in common polymeric materials (e.g., nitrogen)	–	10–200 ng cm^−2^	Wagner et al., 2002
	ToF-SIMS	Good detection limit, high spatial resolution	Limited sampling depth	–	0.1–49 ng cm^−2^	Wagner et al., 2002; Madiona et al., 2017
	Total protein assay (bicinchoninic acid)	Affordable	Requires detergents, large surface areas	–	0.5 ug mL^−1^	Pierce Biotechnology, 2017
	ELISA	Protein type specific, orientation information	Expensive, time consuming	–/+	0.5–5 ng cm^−2^	Ngo and Grunlan, 2017
	LC–MS	Protein specific information	High cost	–	1 pg mL^−2^/2–4 pmol mm^−2^	Rao et al., 2013; Maes et al., 2014
	Coated AFM	Quantitative adhesion force	Tip labeling/modification with proteins is required	–	10 pN	Medalsy et al., 2011
	Fluorescent labeling	Affordable, quantitative	Simple protein mixtures	+	1 ng cm^−2^	Wei et al., 2014
	Radiolabeling	Quantitative, good detection limit, small label size	Handling, accessibility	+	0.05 ng cm^−2^	Felgueiras et al., 2018
Cell adhesion	Visible light microscopy	Common instrumentation, cell geometry (e.g., spreading, elongation)	Rudimentary data provided	–/+	~0.3 μm	Ntziachristos, 2010
	SEM	Direct adhesion visualization	Low throughput, sample prep	–	10 nm	Santoro et al., 2017

*Label required (+), label free (–). SPR, surface plasmon resonance; QCM-D, quartz crystal microbalance with dissipation monitoring; TIRF, total internal reflection fluorescence microscopy; XPS, x-ray photoelectron spectroscopy; ToF-SIMS, time of flight secondary ion mass spectrometry; ELISA, enzyme-linked immunosorbent assay; LC-MS, liquid chromatography-mass spectrometry; AFM, atomic force microscopy; SEM, scanning electron microscopy*.

a *Thickness of overlayer*.

### SPR and QCM-D

Surface plasmon resonance (SPR) allows for the characterization of low-fouling surface coatings, usually polymeric films, with unmodified protein solutions and complex biofluids; SPR signal increases with protein adsorption allowing real-time measurements. SPR is limited to characterizing films that can be synthesized from or grafted to the sensor's gold surface; the immobilized coating must be compatible with flow conditions of the SPR microfluidics. Generally, SPR's limit of detection is reported around 0.3 ng cm^−2^, which is above the fouling limit of several reported low-fouling surfaces, making comparisons difficult for very lower-fouling materials (Blaszykowski et al., 2012). Furthermore, the calculation of total protein non-specifically adsorbed relies on calibration standards that assume saturated monolayers of model proteins (Zhang et al., 2006).

Quartz crystal microbalance with dissipation monitoring (QCM-D) is less sensitive (1.8 ng cm^−2^) (Edvardsson, 2009) than SPR but offers greater variety of sensor surface chemistry, with metallic and polymeric coatings commercially available for functionalization. The added complexity of QCM-D data compared to SPR affords additional capabilities providing insight into the adsorbed protein layer's mechanical properties (Tonda-turo et al., 2018). Furthermore, the sensitivity of QCM-D sensors to changes in the viscoelastic properties of overlayers can be used to characterize changes in cellular dynamics once adhered to a surface of interest (Kushiro et al., 2016). Similar to SPR, low-fouling coatings must be compatible with QCM-D's microfluidic system, and coating thickness may hinder protein adsorption within the detection volume of the sensor (Luan et al., 2017). Unique to QCM-D sensors, the viscosity and thickness of anti-fouling polymer brush layers on QCM-D sensors are known to influence fouling results.

### Ellipsometry

Ellipsometry is a light-based method used to measure film thickness by variations in reflected polarized light and can detect protein adsorption down to 5 ng cm^−2^ with a large array of available surface chemistries for coatings (Welch et al., 2017). Unlike SPR and QCM-D, ellipsometry does not require flow conditions, but sensors must be made from reflective materials for sample characterization in liquid or air. The technique is routinely employed to characterize the modification of materials with polymer overlayers. Ellipsometry measurements can determine adsorbed protein film thickness and mass from refractive index and thickness values (Hook et al., 2001). Ellipsometry has also been combined with other methods, such as QCM-D to provide richer protein adsorption data. On a nanopillar surface, ellipsometry models in conjunction with QCM-D were used to distinguish between fibronectin adsorbed to the tops or in between nanopillars to elucidate how location of adsorbed protein impacts cell adhesion (Kasputis et al., 2015).

### Atomic Force Microscopy

Atomic force microscopy (AFM) can image proteins adsorbed on surfaces, providing protein conformation information. On flat surfaces, cell adhesion and spreading have also been characterized by AFM. For example, AFM was used to determine the conformation of non-specifically adsorbed immunoglobulin G and its impact on *Staphylococcus epidermidis* adhesion (Hou et al., 2018). The conformation of bovine serum albumin (BSA) adsorbed onto surfaces with physiosorbed or covalently bound RGD peptide was also deduced by AFM to demonstrate that BSA conformation is maintained in “ECM-like environments” (Pinho and Piedade, 2013).

## Methods for Measuring Protein Adsorption on Complex Materials

Not all low-fouling materials are amenable to characterization methods utilizing planar sensor surfaces, as described in *Methods for Quantifying Bulk Protein Fouling on Planar Surfaces*. For example, the material may not be amenable to surface grafting or important material properties; such stiffness and surface structures may not be recapitulated on the sensor surface. The following techniques are routinely employed to quantify protein adsorption without planar sensor surfaces. These techniques not only offer greater experimental flexibility and detection specificity but also require the careful selection of controls to ensure results can be accurately interpreted (Tables 2, 3).

**Table 3:** Techniques for the characterization of biological responses to biomaterial surfaces.

**Biological response**	**Technique**	**Advantages**	**Limitations**	**References**
Thrombogenesis	ELISA, Optical density	Recapitulates blood response	Impacted by blood source, storage and test setup	Weber et al., 2018
Platelet activation	ELISA, microscopy	Recapitulates blood response	Impacted by blood source, storage and test set up. Expensive detection	Weber et al., 2018
Macrophage activation/polarization	ELISA, microscopy	Relevance to *in vivo* outcomes	M1–M2 classification may be too simplistic	Brown et al., 2012; Murray et al., 2015
Biofilm formation	Surface culture, microscopy	Relevant, challenging endpoint	Variable with strain and environment	Sjollema et al., 2018

### Methods for Characterizing Unlabeled Proteins

#### Extraction of Adsorbed Protein for Quantification

After materials are exposed to protein solutions or biological fluids, unlabeled proteins are removed from the surface of interest with a detergent compatible with total protein detection assay such as sodium dodecyl sulfate (Ju et al., 2009; Ma et al., 2015; Dong et al., 2016). For example, the bicinchoninic acid (BCA) assay detects protein peptide bonds and has been used for adsorbed protein quantification on a variety of biomaterials, with detection levels of ~ 1 μg cm^−2^. The absorbance signal produced by the BCA assay is amino acid dependent; calibration curves should therefore be prepared with proteins of interest (Walker, 1996).

Methods requiring extraction of adsorbed proteins are limited by the fact that most detergents do not quantitatively remove all proteins from surfaces (Riedel et al., 2016). Therefore, assays usually report values relative to positive controls; calibrations curves can estimate adsorbed protein amounts, assuming near quantitative removal (Jesmer et al., 2020). The use of detergents prevents the investigation of adsorbed protein conformation, or bioactivity.

#### Enzyme-Linked Immunosorbent Assay: Detecting Adsorbed Proteins

Enzyme-linked immunosorbent assays (ELISA) can be used to measure proteins non-specifically adsorbed to surfaces, blood complement activation, and proteins produced by adhered cells. ELISAs detect surface adsorbed proteins, which act as the capture layer. Generally, ELISAs are limited to detecting a single adsorbed protein; ELISAs are therefore suitable for fouling experiments using a simple protein solution (i.e., fibronectin solution) or a biofluid to detect a specific protein's adsorption from a complex mixture (i.e., fibrinogen adsorption from blood).

ELISA can provide adsorbed protein conformation and bioactivity information. ELISAs have been used to measure adsorbed fibronectin bioactivity, which is advantageous over total protein measurements that cannot assess bioactivity (Tziampazis et al., 2000; Seo et al., 2013). ELISAs can also be used to detect potential immune responses. To measure complement activation due to a hydrogel, an ELISA was used to measure C5b-9 complement activation in serum exposed to material surfaces (Li B. et al., 2019).

ELISA measurements are independent of the substrate material, eliminating the need for proxy surfaces like SPR's gold sensors. When measuring fibrinogen adsorption onto antifouling zwitterionic coatings on planar materials, ELISA and SPR were compared (Cheng et al., 2009). Upon quantifying fibrinogen adsorption, ELISA indicated greater adsorption than SPR due to differences in polymer grafting density between glass and the SPR gold sensor. Therefore, ELISAs may provide more relevant data for non-gold surfaces.

#### Liquid Chromatography With Mass Spectrometry: Determining Adsorbed Protein Content After Extraction

Understanding the types and ratios of adsorbed proteins may provide insight into potential downstream *in vivo* effects and immune responses upon implantation (Othman et al., 2018). To profile all adsorbed proteins, liquid chromatography with mass spectrometry (LC-MS) may be employed to provide more information than total protein methods such as BCA. For example, protein adsorption from serum onto surfaces of varying hydrophilicity (water contact angle of 49° to 92°) showed similar total protein levels on all surfaces, but LC-MS determined differences in the types of proteins adsorbed. The same surfaces also displayed different bioactivities, which was demonstrated by tracking cytokine release from macrophages seeded on the biomaterial surface; macrophages released more proinflammatory cytokines [tumor necrosis factor α (TNF-α), interleukin 6 (IL-6), IL-1b, interferon γ-induced protein 10] and less anti-inflammatory cytokines (arginase, IL-10) with increasing surface hydrophobicity (Visalakshan et al., 2019). Protein profiling provided key information that total protein characterization could not provide.

LC-MS protein identification has been used to relate adsorbed proteins on hydrogel implants to the potential FBR capsule formation and thickness. On hydrogels that varied in composition and stiffness, total adsorbed protein amounts did not correlate to FBR capsule thickness. LC-MS analysis of adsorbed proteins 30 min after implantation demonstrated that the presence of proteins associated with extracellular matrix construction and cell adhesion were strong predictors of FBR capsule thickness (Jansen et al., 2018).

#### Surface Sensitive Techniques: X-Ray Photoelectron Spectroscopy and Time of Flight Secondary Ion Mass Spectrometry

Surface sensitive techniques to determine material composition can characterize protein overlayers on biomaterials. Both x-ray photoelectron spectroscopy (XPS) and time of flight secondary ion mass spectrometry (ToF-SIMS) detect only the first ~10 and 2 nm, respectively, of a material (Castner and Ratner, 2002), making them ideal techniques for quantification of adsorbed material without requiring extraction and collection. Although substrate composition (e.g., elemental composition overlap with protein) and film thickness impact the sensitivity of both techniques (Wagner et al., 2002). Film thickness impacts each technique differently as the sampling depth of XPS is deeper than ToF-SIMS, for example, on sodium styrenesulfonate-coated and bare gold surfaces exposed to various protein solutions, ToF-SIMS signals saturated before XPS signals of protein adsorption, due to the increased sampling depth of the XPS technique as adsorbed protein overlayers can be thicker than the ToF-SIMS sampling depth (Foster et al., 2016). XPS and ToF-SIMS have been used in conjunction to measure surface chemical composition and protein adsorption levels on gradient polyethylene glycol (PEG) surfaces. The high spatial resolution of the techniques and ability of both to detect protein *in situ* allowed for correlation between adsorbed protein and surface polymer density without requiring multiple sample surfaces (Menzies et al., 2012).

### Methods for Quantifying Adsorbed Proteins Modified With Detection Labels

Quantification of fluorescent or radioactive protein is easily achieved using the corresponding instrumentation with standards for calibration; labeled proteins have improved limits of detection when compared to absorbance-based protein quantification methods (Table 2). Labels may limit fouling studies to individual proteins and alter protein properties such a hydrophobicity and bioactivity. Generally, *in vitro* fouling assays with labeled protein are carried out below physiological concentrations without competing proteins, which may poorly predict *in vivo* performance. Therefore, fluorescent and radioactive labels offer greater sensitivity, but current experimental design for fouling experiments may not always mimic *in vivo* conditions.

#### Fluorescent Labels

Because of their high sensitivity, fluorescently labeled proteins are commonly used to characterize non-specific protein adsorption to surfaces. Quantification of adsorbed fluorescent proteins is regularly performed by fluorescence microscopy or protein extraction for solution fluorescent measurements.

Fluorescent techniques can also provide information about protein folding, orientation, and reversibility of non-specific adsorption at single-molecule binding resolution. Using fibronectin with fluorescence resonance energy transfer (FRET) labels, residence time and folding state of adsorbed protein were determined on different polymeric PEG surfaces. Fibronectin's adsorption rate decreased with higher PEG density, but surface residence time increased because of more protein unfolding (Marruecos et al., 2016); greater residence time with higher PEG densities has not been observed with unlabeled proteins. Fluorescein, a commonly used fluorescent label, was shown to change the orientation of adsorbed lysozyme on surfaces in a modeling study (Romanowska et al., 2015). FRET pairs have also been used to confirm conformation of surface adsorbed protein (Marruecos et al., 2016). Single-molecule resolution TIRF for Alexa Fluor 647–labeled BSA and fibrinogen fouling on PEG surfaces determined that non-specific adsorption was reversible. Fouling was also inversely correlated to protein concentration on PEG surfaces, where ~1 μg mL^−1^ BSA and fibrinogen solutions resulted in fouling, whereas the use 1 mg mL^−1^ solutions yielded surfaces with no detectable protein (Hedayati et al., 2020). The authors proposed that increased protein concentrations near the material surface could stabilize protein conformation and decrease the likelihood of proteins denaturing on the surface leading to lower fouling levels at the higher concentration.

Fluorescent labels can also alter protein properties that may influence adsorption degrees. For example, fluorescent labeling has been shown to influence the protein's isoelectric point (by ~0.1), size, and charge (Bingaman et al., 2003). Characterization via single-labeled fluorescent proteins does not provide information on protein conformation or orientation and is limited to simple protein solutions; the use of biofluids is difficult because of labeling differences within the large protein population.

#### Radioactive Labels

In contrast to fluorescent tags, radioactive labels can be introduced during protein expression with a radioactive amino acid or by covalently grafting a small tag. Because of high sensitivity, radio-tagged protein assays have detection limits as low as 0.05 ng cm^−2^ (Felgueiras et al., 2018). While grafting small radiolabels has minor impacts on protein properties, oxidizing conditions used in many labeling reactions can result in protein aggregation and degradation, resulting in greater protein adsorption over unlabeled proteins (Holmberg et al., 2007). Radiolabels are typically used to measure total adsorption of an individual protein and do not provide information about protein conformation or orientation.

#### Coated AFM: Modified Tips for Selective Protein Detection

Protein-coated AFM tips can quantify adhesive forces between individual adsorbed proteins and biomaterials, which can impact cell adhesion outcome. Fibronectin-coated AFM in concert with fluorescently labeled proteins can be used to correlate total protein adsorption (via fluorescence) and adhesive forces to surfaces; a strong linear relationship between single protein adhesive strength and total protein adsorption is usually observed (Taylor et al., 2008). In conjunction with ELISA, AFM can be used to measure protein adsorption force to multiple surface chemistries and protein conformation. Fibronectin-coated AFM also demonstrated that the strength of protein material interactions determines cell fate. Stronger interactions between fibronectin and materials led to decreased cell viability by hindering matrix remodeling (González-garcía et al., 2018).

## Cellular Adhesion and Activation

For biomaterials that will be exposed to cells, quantifying cell adhesion and activity is necessary as protein adsorption does not necessarily correlate with downstream cellular activities; even when protein adsorption is below detection limits, cells have been shown to interact with surfaces.

Low-fouling materials are generally designed to prevent or minimize cell adhesion, but cell adhesion can be advantageous for some medical implants. For example, adhesion of cells associated with anti-inflammatory pathways may improve biomaterial outcomes, and cell integration is necessary for dental and joint replacements, although cellular interactions with biomaterials should be studied to avoid deleterious immune responses for most medical implants.

### Quantifying Mammalian Cell Fouling

Interactions between mammalian cells and biomaterials are routinely characterized by (1) detection of the adhered cells through microscopy or metabolic activity and (2) detection of signals produced by cells (i.e., adhesins or cytokines; Tables 2, 3). These methods are complementary and together can provide detailed information regarding biomaterial fouling and potential immune outcomes. *In vitro* methods to recapitulate the full *in vivo* immune response remain an active area of research (Sharifi et al., 2019).

Adhered cells are commonly characterized by microscopy to determine cell number, morphology, elongation, and spreading, which can all be related to cell bioactivity. For example, cell morphology has been linked to macrophage phenotype, with elongated cells exhibiting anti-inflammatory properties (Luu et al., 2015). SEM and fluorescence microscopy have also been used to quantify cell elongation and spreading on grooved surface, which correlated with cytokine profiles (Luu et al., 2015). To study interactions between topographical surfaces with cells, a method combining focused ion beam and SEM (FIB-SEM) was developed to determine cell adhesion preferences and morphologies as a function of nanostructures. Cells were found to preferentially bind to protrusions over pores by visualizing adhesion points (Santoro et al., 2017).

Biochemical techniques used in concert with microscopy can find trends between bioactivity and cell number or morphology. ELISA assessment of IL-6 and TNF-α with fluorescent microscopy demonstrated that macrophage adhesion on fibronectin-coated surfaces correlated with a low inflammatory activation state; FRET experiments indicated that cells on surfaces with stabilized fibronectin had low inflammatory cytokine profiles (Faulón Marruecos et al., 2019). Interestingly, unfolded adsorbed fibronectin promoted a proinflammatory state, indicating the need to study protein stability and not only total amounts.

The treatment and preparation of materials before cell adhesion assays can impact cell adhesion outcomes; pre-exposing biomaterial surfaces to proteins prior to cell assay impacts cell density and spreading (Jansen et al., 2018). For example, protein choice during pre-exposure impacted the adhesion and spreading of human fibroblasts on polymer coated titanium surfaces; BSA did not significantly influence cell adhesion or spreading unlike fibrinogen, which promoted adhesion and spreading (Pei et al., 2011).

#### High-Throughput Methods to Measure Macrophage Adhesion and Activation

To minimize proinflammatory polarization of immune cells, implantable biomaterials are now being designed to promote anti-inflammatory polarizations. To this end, a high-throughput method for non-specific protein adsorption alongside macrophage adhesion and polarization, a component of inflammation (Brown et al., 2012), was developed using microprinted polymer spot arrays (Rostam et al., 2020). Polymer spot microarrays were assayed for cell attachment and macrophage polarization by microscopy and calprotectin/mannose receptor staining. HTS hits were then subjected to more rigorous screens for cytokine profile and phagocytic ability of macrophages as well as mass spectrometry of adsorbed proteins from fetal bovine serum.

### Bacterial Fouling Related to Medical Implants

Resistance of material surfaces to bacterial colonization is commonly pursued through two main strategies of (1) adhesion resistance and (2) active killing. Adhesion resistance strategies prevent bacteria from adhering and eventually forming biofilms, usually through methods that repel protein and host cell adhesion. In active killing strategies, surfaces may kill settled bacteria on contact through chemical or physical means or the release antibacterial agents (Campoccia et al., 2013). Methods of testing *in vitro* bacterial fouling have been well reviewed recently (Azeredo et al., 2017; van de Lagemaat et al., 2017; Boudarel et al., 2018; Sjollema et al., 2018).

When measuring the resistance of a biomaterial to bacterial adhesion and biofilm formation, the environment of the intended implant location should be replicated. The implant site will also guide the selection of bacteria strain to investigate. Implant sites also vary in shear forces from fluid flow, immune environments, and host cell–bacteria interactions (Busscher et al., 2012) (Table 4). For example, two low fouling surfaces with similar resistance to fibrinogen adsorption showed drastically different biofilm formation when exposed to the *P. aeruginosa* due to differences in flow conditions; under static conditions, no biofilm was observed after 6 months (Wang et al., 2020), whereas biofilms formed after only 10 days under flow conditions (Cheng et al., 2009).

**Table 4:** Primary bacterial infections and conditions by implant site.

**Implant site**		**Primary bacterial infection**	**Shear rate (s^**−1**^)**	**Fluid type**	**REF**
Ocular surface		*P. aeruginosa*/*S. epidermidis*	0.35	Tears	Bakker et al., 2003; Dutot et al., 2009
Urinary tract		*P. aeruginosa*	15	Urine	Dohnt et al., 2011; Azevedo et al., 2017
Bone		*S. aureus*/CoNS	—	—	Li and Webster, 2017
Spinal column		Early *S. aureus*, late *P. acnes*	—	—	Lall et al., 2015
Peritoneal cavity		*S. epidermidis*/*S. aureus*	20–120	CSF	Bloomfield et al., 1998; Vinchon and Dhellemmes, 2006
Vascular	TIVAP	<30 d—*S. aureus* Total—CoNS	10–1,000Casa et al., 2015	Blood	Lebeaux et al., 2014
	Vascular graft	Early CoNS, Late *S. aureus*/*E. coli*			Saleem et al., 2010
	PICC/CVC	CoNS			Haddadin et al., 2020

*CoNS, coagulase-negative staphylococci; CSF, cerebrospinal fluid; TIVAP, totally implantable venous access port; PICC, peripherally inserted central venous catheter; CVC, central venous catheter; early, <3 months after surgery; late, >3 months after surgery*.

### Tissue and Bacterial Cell Co-culture

Implanted materials that lead to infection will be in the presence not only of bacterial cells but also of the host tissue, both of which compete for the implant surface (Busscher et al., 2012). Co-cultures of bacterial and mammalian cells have therefore been used, especially with respect to bacterial effects on implant integration. Co-culture setup requires preliminary work with monoculture to determine optimal growth densities and conditions that will support both cell types (Zaatreh et al., 2016). In addition to live bacterial strains, co-culture setups including heat-killed bacteria have been used to study the influence of the presence of bacteria without requiring conditions that support both cell types. A study including heat-killed bacteria found that presence of low levels of bacterial signals could improve integration of biomaterials, although the authors highlight the limitations of their results to the specific experimental setup (Yue et al., 2015). Bacterial response to biomaterials are dictated by their environment leading to different outcomes when cultured alone or in the presence of eukaryotic cells; for example, surfaces exposed to mammalian cells may have antimicrobial activity by enhancing immune responses directed toward bacteria (Li J. et al., 2019; Yang et al., 2019).

### Choosing Relevant Fouling Assays

The intended application will dictate the *in vitro* biofouling assay(s) required to assess biomaterials, with the site and length of implantation being prominent factors to select protein or biofluid sources, concentrations, cell types, flow rates and fluid-associated stress, and biological characterizations (e.g., cytokines related to immune responses). Immune responses are more important for long-term implants, and cell types should be chosen to investigate long-term biological responses such as the fibrotic response. Furthermore, some biomaterials require tissue integration (e.g., dental implant, joint replacement), whereas others benefit from the lack of tissue integration (e.g., catheters), which will also help identify requirements for *in vitro* biofouling experiments.

**Figure 3:**
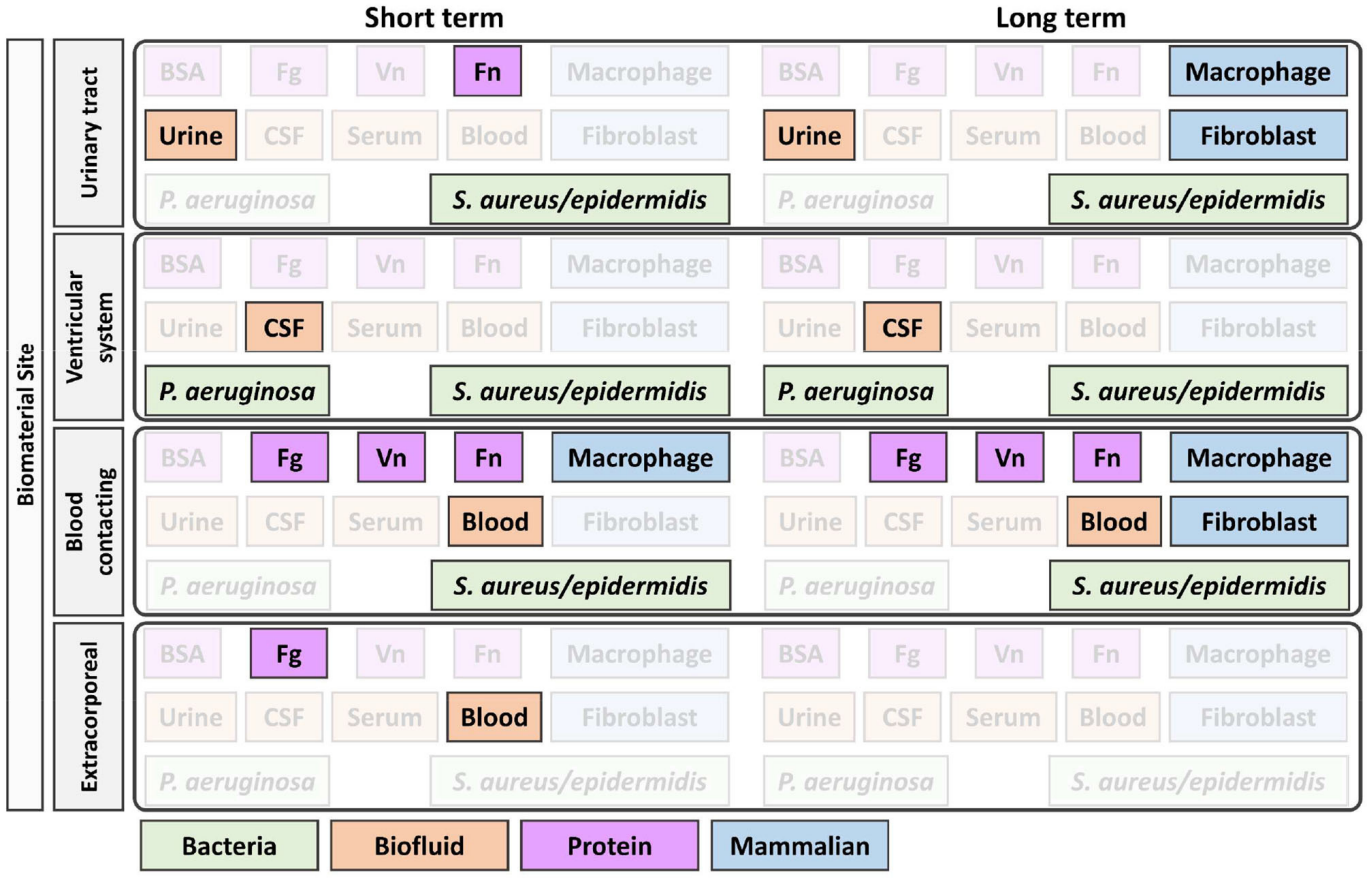
Identification of appropriate *in vitro* biofouling experiments for low-fouling biomaterials. The site and length of implantation will determine the variety of biological components for screening. Above is an example of how the site and length of implantation can help guide the selection of biologic components for biofouling experiments. For experimental setup consideration of proteins and cells, refer to Figures 1, 2, respectively. BSA, bovine serum albumin; Fg, fibrinogen; Vn, vitronectin; Fn, fibronectin; *P. aeruginosa, Pseudomonas aeruginosa*; *S. aureus*/*epidermidis, Staphylococcus aureus/Staphylococcus epidermidis*.

In combination with Figures 1–3 was designed to help guide the selection of biofouling experiments, although each material in development will need to be carefully considered to select appropriate biofouling experiments and controls. Figure 3 highlights the most common biofouling tests required for biomaterials as a function of duration of implantation (short vs. long term) and the site of biomaterial implantation (biomaterial site). Once the protein and cell fouling experiments are identified, experimental design must be carefully executed to establish *in vitro* conditions that mimic *in vivo* fouling, which is explained in Figures 1, 2.

Despite being an affordable and accessible protein, BSA is not recommended for any specific implantation site in Figure 3 as the protein is not physiologically relevant and differs in sequence and fouling profile, even compared to human serum albumin (Su et al., 2000). Blood serum is a commonly used and accessible biological material used for fouling experiments, but limitations in its applicability should be considered when planning fouling experiments. Blood serum is free of fibrinogen, which was found to adsorb to all tested low-fouling PEG-based surfaces (Riedel et al., 2013), and the age of serum samples lead to different fouling profiles (Yang et al., 2009). Further, serum properties are not well-defined and vary by batch and supplier, and moreover, there are interspecies differences between the commonly used fetal bovine serum and human serum, which alter cell behavior *in vitro* (Heger et al., 2018).

Following the choice of appropriate biological material and conditions, techniques should be chosen that can measure the outcomes of biofouling in different ways to internally corroborate findings. More than one technique should be used to characterize biofouling in order to overcome the limitations of individual techniques (Tables 2, 3), increase confidence in results, and improve ability to compare findings between papers. To further improve interstudy comparability, standard negative and positive controls such as glass and tissue culture plastic should be included in each *in vitro* assay; with consistent surface and experimental controls within the literature, it will improve our ability to compare published data to better interpret the antifouling properties of new materials. The same material should be used throughout characterization steps; for example, if a polymer coating is being characterized, the substrate material should be the same for all tests to control for differences in grafting density and stiffness. Finally, experimental details and the reasons for their choice should be clearly stated, and their impact on results discussed as outlined in the MIRIBEL standard (Faria et al., 2018).

## Conclusions

The discovery and development of low-fouling or controlled fouling biomaterials remain an important active area of research. The current heterogeneity of *in vitro* biofouling experimental conditions and characterization methods hinders biomaterial discovery and translation. This review highlights how controls, experimental design, and characterization limitations must be considered for the interpretation of *in vitro* results. Ideally, *in vitro* conditions will mimic conditions for the biomaterial's intended application to accelerate translation into the real world and clinic. To this end, the review provides guidance for the selection and execution of *in vitro* fouling experiments, while stressing that the biomaterial under study will greatly influence the needed biofouling experimentation.

## Summary

Understanding the extent and nature of biomaterial fouling is crucial for the development of medical devices and antibacterial surfaces. In light of recent insights into protein–material interactions and heterogeneity of reported fouling values, we review common experimental conditions and detection methods for *in vitro* fouling measurements to establish guidelines for improved *in vitro* fouling experiments. The ultimate goal is to expedite the discovery of low-fouling biomaterials.

## Author Contributions

AJ and RW conceived, wrote, and prepared the manuscript. All authors contributed to the article and approved the submitted version.

## Conflict of Interest

The authors declare that the research was conducted in the absence of any commercial or financial relationships that could be construed as a potential conflict of interest.
